# Distinct Analgesic Actions of DHA and DHA-Derived Specialized Pro-Resolving Mediators on Post-operative Pain After Bone Fracture in Mice

**DOI:** 10.3389/fphar.2018.00412

**Published:** 2018-05-01

**Authors:** Linlin Zhang, Niccolò Terrando, Zhen-Zhong Xu, Sangsu Bang, Sven-Eric Jordt, William Maixner, Charles N. Serhan, Ru-Rong Ji

**Affiliations:** ^1^Department of Anesthesiology, Center for Translational Pain Medicine, Duke University Medical Center, Durham, NC, United States; ^2^Department of Physiology, Center of Neuroscience, Key Laboratory of Medical Neurobiology of the Ministry of Health of China, Zhejiang University School of Medicine, Hangzhou, China; ^3^Department of Anesthesiology, Center for Experimental Therapeutics and Reperfusion Injury, Perioperative and Pain Medicine, Brigham and Women’s Hospital, Harvard Medical School, Boston, MA, United States; ^4^Department of Neurology, Duke University Medical Center, Durham, NC, United States

**Keywords:** DHA (docosahexaenoic acid), fPOP (post-operative pain after bone fracture), omega-3 poly unsaturated fatty acids, orthopedic surgery, post-surgical pain, spinal cord, SPMs (specialized pro-resolving mediators)

## Abstract

Mechanisms of pain resolution are largely unclear. Increasing evidence suggests that specialized pro-resolving mediators (SPMs), derived from fish oil docosahexaenoic acid (DHA), promote the resolution of acute inflammation and potently inhibit inflammatory and neuropathic pain. In this study, we examined the analgesic impact of DHA and DHA-derived SPMs in a mouse model of post-operative pain induced by tibial bone fracture (fPOP). Intravenous perioperative treatment with DHA (500 μg), resolvin D1 (RvD1, 500 ng) and maresin 1 (MaR1, 500 ng), 10 min and 24 h after the surgery, delayed the development of fPOP (mechanical allodynia and cold allodynia). In contrast, post-operative intrathecal (IT) administration of DHA (500 μg) 2 weeks after the surgery had no effects on established mechanical and cold allodynia. However, by direct comparison, IT post-operative treatment (500 ng) with neuroprotectin D1 (NPD1), MaR1, and D-resolvins, RvD1 and RvD5, but not RvD3 and RvD4, effectively reduced mechanical and cold allodynia. ELISA analysis showed that perioperative DHA treatment increased RvD1 levels in serum and spinal cord samples after bone fracture. Interestingly, sham surgery resulted in transient allodynia and increased RvD1 levels, suggesting a correlation of enhanced SPM levels with acute pain resolution after sham surgery. Our findings suggest that (1) perioperative treatment with DHA is effective in preventing and delaying the development of fPOP and (2) post-treatment with some SPMs can attenuate established fPOP. Our data also indicate that orthopedic surgery impairs SPM production. Thus, DHA and DHA-derived SPMs should be differentially supplemented for treating fPOP and improving recovery.

## Introduction

Nerve injury-induced neuropathic pain due to diabetic neuropathy, viral infection, and chemotherapy is a major health problem worldwide (Woolf and Mannion, 1999; Dworkin et al., 2003; Campbell and Meyer, 2006; Kehlet et al., 2006). Similarly, common major surgeries frequently lead to the development of chronic post-operative pain (POP; Kehlet et al., 2006). Orthopedic injuries and subsequent surgery, such as tibial fracture and repair, produce sustained POP for many weeks that can greatly affect the quality of life in susceptible patients. Fracture associated POP (fPOP) is related to nerve injury, as reflected by a robust induction of the transcription factor ATF3 in sensory neurons, as well as neuroinflammation in the peripheral and central nervous system (Li et al., 2015; Zhang et al., 2016). Over the past three decades, great progress has been made in clarifying the mechanisms underlying the pathogenesis of pain after inflammation and nerve injury. It is generally accepted that pathological pain is caused by neural plasticity in primary sensory neurons (peripheral sensitization), spinal cord dorsal horn, and brain neurons (central sensitization; Gold and Gebhart, 2010; Woolf, 2011; Ji et al., 2018). Dysregulation of glial cells (gliopathy) also contributes to the pathogenesis of pain in part by promoting neuroinflammation (McMahon and Malcangio, 2009; Ji et al., 2013, 2018; Tsuda, 2017).

Despite our progress in understanding the induction mechanisms producing the perception of pain (Ji et al., 2011), failure to resolve acute pain may in fact lead to the transition to chronic and maladaptive pain states (Ji et al., 2011; Willemen et al., 2014). Serhan and his coworkers have demonstrated that the resolution of acute inflammation is an active process and requires biosynthesis of specialized pro-resolving mediators (SPMs). SPMs, such as resolvins, lipoxins, neuroprotectins, and maresins, are derived from omega-3 polyunsaturated fatty acids docosahexaenoic acid (DHA) and eicosapentaenoic acid (EPA) and exhibit potent anti-inflammatory and pro-resolution actions in various animal models of inflammation (Serhan et al., 2008; Norling et al., 2016). Many groups have demonstrated that peripheral, spinal, or systemic administration of lipoxins (LXA_4_), resolvins such as RvD1, D2, and E1 (RvD1, RvD2, and RvE1), NPD1, and MaR1 at very low doses (nanogram range), effectively reduced inflammatory pain (Svensson et al., 2007; Bang et al., 2010; Xu et al., 2010; Lima-Garcia et al., 2011; Park et al., 2011a,b; Sommer and Birklein, 2011; Terrando et al., 2013), POP after thoracotomy and muscle retraction (Huang et al., 2011; Wang and Strichartz, 2017), as well as neuropathic pain after nerve injury (Xu et al., 2013a,b) and spinal cord injury (Martini et al., 2016).

Although SPMs have been tested in several animal models of persistent pain, the following issues remain to be addressed: (1) the unique role of different SPMs in fPOP has not been investigated; (2) RvD3, RvD4, and RvD5 are identified new members of resolvin family and their complete stereochemical structures and total organic synthesis were recently achieved (Serhan et al., 2002; Dalli et al., 2013; Norris et al., 2016, 2017; Winkler et al., 2016, 2018), but their involvement in pain is unexplored; (3) it is unclear whether SPMs can be produced and converted, under surgical manipulations (sham surgery vs. bone fracture) from their fish oil precursor DHA; and (4) it is also unclear if SPMs and their precursor (fish oil) exert differential effects in preventing or treating/reversing pathological pain. The present study was designed to address these questions by focusing on DHA and DHA-derived SPMs (RvD1, RvD3, RvD4, RvD5, NPD1, and MaR1) in a clinically relevant surgical pain model. Our results demonstrate unique analgesic effects of DHA and SPMs in preventing and reversing fPOP, revealing distinct production of SPMs after sham surgery and bone fracture.

## Materials and Methods

### Animals and Surgery

Adult CD1 mice (male, 25–35 g) were purchased from Charles River Laboratories. All animal procedures performed in this study were approved by the Animal Care Committee of Duke University and the ethics guidelines of International Association for the Study of Pain were followed (Zimmermann, 1983). Tibial facture was performed under isoflurane anesthesia as we described previously (Zhang et al., 2016; Luo et al., 2017). Muscles were disassociated following an incision on the left hind paw. A 0.38-mm stainless steel pin was inserted into the tibia intramedullary canal, followed by the osteotomy. The incision was sutured with 6-0 Prolene.

### Drugs and Administration

Docosahexaenoic acid, RvD1, RvD5, and MaR1 were purchased from Cayman Chemicals. RvD3 and RvD4 were validated with authentic standards (Serhan Lab). NPD1 was a kind gift from Resolvyx Pharmaceuticals, Inc. (Cambridge, MA, United States). For perioperative treatment, DHA (500 μg, 100 μl) or SPMs (500 ng, 100 μl) were dissolved in 2% ethanol as vehicle and administered intravenously through tail vein injection at 10 min and 24 h after surgery. For intrathecal (IT) injection, spinal cord puncture was made with a 30G needle between the L5 and L6 levels to deliver reagents (10 μl, 500 μg DHA or 500 ng SPMs, dissolved in 10% ethanol) into the cerebral spinal fluid (Hylden and Wilcox, 1980). Intravenous or IT injections were given under brief isoflurane anesthesia.

### ELISA Analysis

Mouse RvD1 ELISA kit was purchased from Cayman Chemicals (Catalog number 500380). The detection sensitivity of this ELISA kit is 15 pg/ml, where is sufficient to detect RvD1 levels in our samples. Serum, spinal cord, and brain tissues were collected from animals before and 5 days after the tibia surgery. Spinal cord and brain tissues were homogenized in a lysis buffer containing protease and phosphatase inhibitors (Zhuang et al., 2006). Tissue samples were centrifuged at 12,500 × *g* for 10 min and the supernatant was collected. Protein concentrations were determined by BCA Protein Assay (Pierce). For each reaction in a 96-well plate, 100 μg of proteins of brain and spinal cord samples and 25 μl of serum were used. ELISA was performed according to the manufacturer’s protocol. The samples and the competition RvD1 tracer [RvD1 linked to acetyl-cholinesterase (AChE)] were incubated overnight at 4°C. The signal in ELISA plate was developed by Ellman’s Reagent, a substrate of AChE. The optical densities of samples were measured using an ELISA plate reader (Bio-Rad) at a wavelength of 412 nm and RvD1 levels were calculated using the standard curves. The standard curve was included in each experiment. The RvD1 values of the samples were in the linear range of the standard curves.

### Behavioral Analysis

Animals were habituated to the testing environment daily for at least 2 days before starting baseline assessment. The room temperature and humidity remained stable throughout the experiments. For testing mechanical sensitivity, animals were put in boxes on an elevated metal mesh floor and allowed 30 min for habituation before examination. The plantar surface of each hind paw was stimulated with a series of von Frey hairs with logarithmically incrementing stiffness (0.02–2.56 g, Stoelting), presented perpendicularly to the plantar surface. Mechanical allodynia was assessed by the frequency response to a sub-threshold low force von Frey hair. Hind paws were stimulated with a 0.16 g von Frey hair for 10 times and the percentage withdrawal response was calculated as frequency (Luo et al., 2017). To assess cold allodynia, two acetone applications (20 μl each) were gently applied to the hind paw bottom using a pipette and the responses to acetone were scored as: 0, no response; 1, quick withdrawal, paw stamping, or flicking; 2, prolonged withdrawal or repeated flicking of the paw; and 3, repeated paw flicking and licking (Han et al., 2016). The experimenter was blinded to the treatments.

### Quantification and Statistics

All data were expressed as mean ± SEM. Statistical analyses were completed with Prism GraphPad 6.0. Differences between groups were compared using two-way or one-way ANOVA followed by Bonferroni *post hoc* test. The criterion for statistical significance was *P* < 0.05.

## Results

### Sham Surgery Produces a Transient Post-operative Pain That Resolves in a Week

**FIGURE 1:**
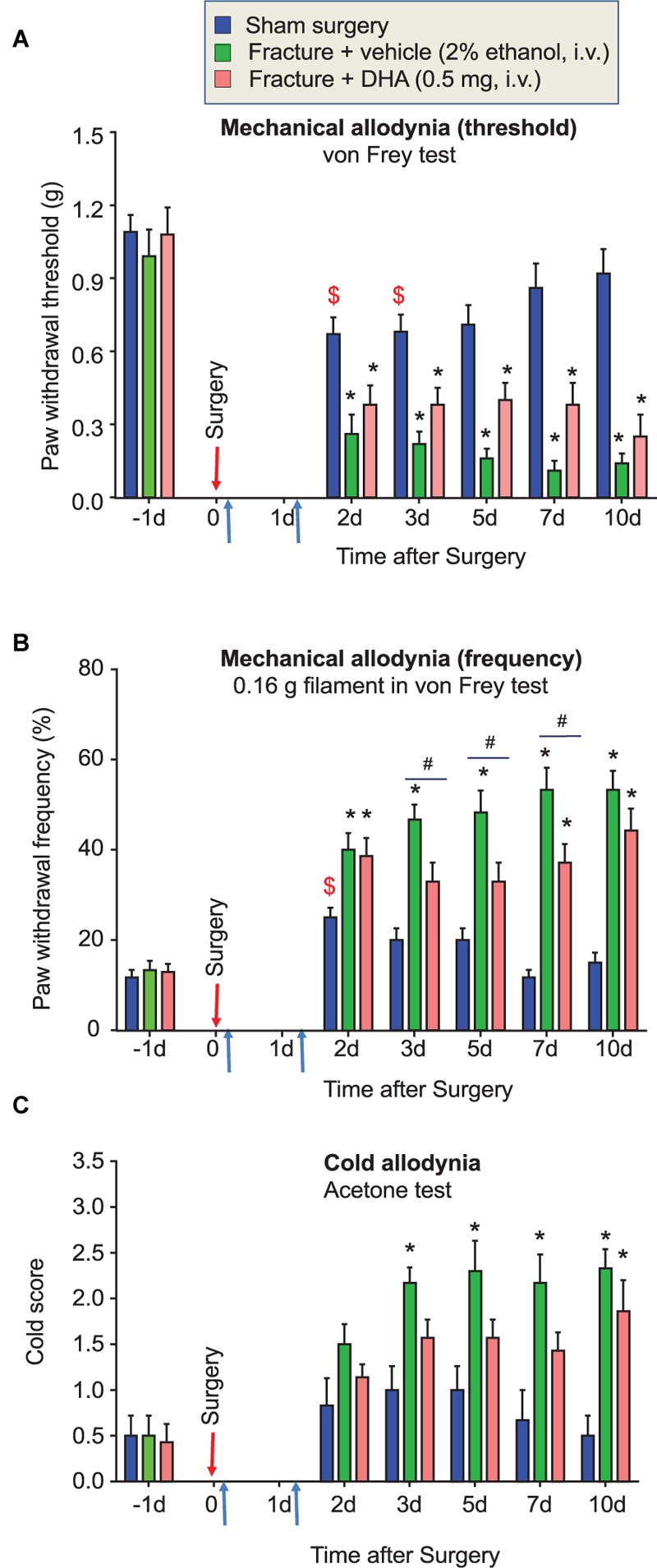
Effects of sham surgery, bone fracture, and perioperative DHA treatment on the development of fPOP. Development of mechanical allodynia, assessed by paw withdrawal threshold **(A)** and paw withdrawal frequency to 0.16 g filament **(B)** in von Frey test, after sham surgery, tibial bone fracture, and perioperative treatment of DHA (0.5 mg, 100 μl, i.v.), given 10 min and 24 h after bone fracture surgery (indicated by blue arrows). **(C)** Development of cold allodynia, assessed by cold response scoring in the acetone test, after sham surgery, tibial bone fracture, and the perioperative DHA treatment after bone fracture surgery (indicated by blue arrows). $*P* < 0.05, one-way ANOVA in Sham group vs. baseline; ^∗^*P* < 0.05 vs. Sham surgery; #*P* < 0.05, fracture vs. fracture + DHA; two-way ANOVA followed by Bonferroni test, *n* = 6–7 mice/group. Data are presented as mean ± SEM.

We first examined mechanical and cold hypersensitivity in sham surgery mice subjected to skin and muscle incision but no pin insertion and bone fracture. Von Frey testing revealed a mild and transient (<7 days) reduction in paw withdrawal threshold in the sham animals. This result suggests that sham surgery procures transient mechanical allodynia, and this acute pain resolves within a week (*P* < 0.05, one-way ANOVA, **Figure 1A**). We also assessed mechanical allodynia measuring frequency responses to a subthreshold von Frey filament (0.16 g) and observed a transient increase (<7 days) in paw withdrawal frequency (*P* < 0.05 vs. baseline, one-way ANOVA, **Figure 1B**). The acetone test revealed that sham surgery also caused a slight increase in cold response scores for 5 days (*P* > 0.05, one-way ANOVA, **Figure 1C**). Together, these results indicate that sham surgery produces transient mechanical and cold allodynia. Thus, this acute surgical pain model can serve as an animal model of pain resolution.

### Tibia Fracture Produces Persistent fPOP, Which Is Partially Prevented by Systemic Perioperative Treatment of DHA

Compared to sham surgery, tibial bone fracture induced persistent fPOP, as revealed by persistent mechanical allodynia (*P* < 0.05), i.e., decrease in paw withdrawal threshold (**Figure 1A**) and increase in paw withdrawal frequency to a sub-threshold von Frey filament (0.16 g), which would not elicit pain under the normal conditions (**Figure 1B**). fPOP also manifested a persistent cold allodynia using the acetone test (**Figure 1C**, *P* < 0.05). Next, we investigated whether perioperative administration of DHA, at 10 min and 24 h after the surgery, would protect from fPOP. Notably, intravenous injections of DHA (500 μg, 100 μl) significantly attenuated mechanical allodynia (*P* < 0.05 vs. vehicle control) by decreasing paw withdrawal frequency (**Figure 1B**). Compared to vehicle control, cold allodynia was not significantly reduced (*P* > 0.05) by DHA (**Figure 1C**). However, cold allodynia in the treatment group was also not significantly different from sham surgery, suggesting a possible inhibition of cold allodynia by the DHA pre-treatment (**Figure 1C**).

### Sham Surgery but Not Orthopedic Surgery Increases RvD1 Levels in Serum and Spinal Cord

**FIGURE 2:**
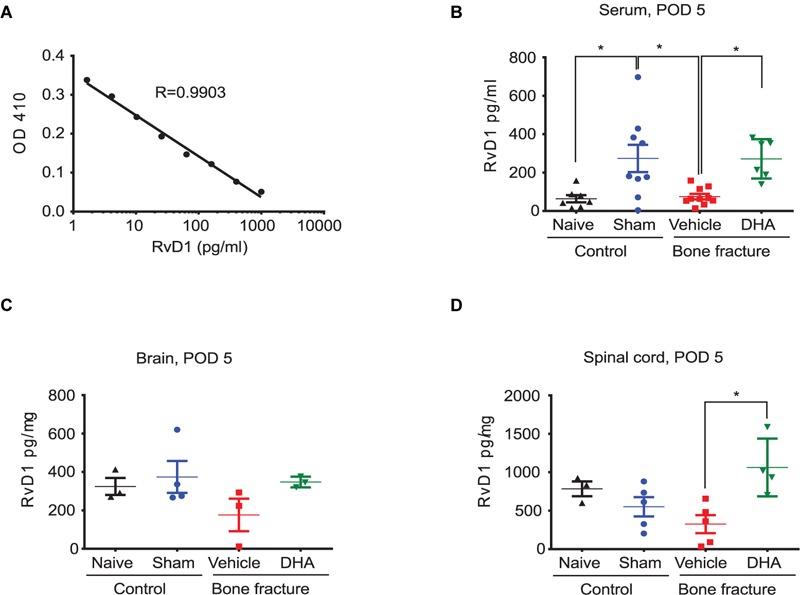
Perioperative DHA treatment increases RvD1 levels in serum and spinal cord. **(A)** RvD1 standard curve produced by the Cayman Chemical ELISA assay, demonstrating reliable measurements within the specified concentration range. RvD1 levels in serum samples **(B)**, brain samples **(C)**, and spinal cord samples **(D)** of naïve mice and mice after sham surgery or bone fracture with vehicle and DHA treatment (the same as described in **Figure 1**). Samples were collected at post-operative day 5 (POD 5). Note that RvD1 levels are elevated after sham surgery and in serum and spinal cord samples after the DHA treatment. ^∗^*P* < 0.05, one-way ANOVA; *n* = 3–10 mice per group. Data are presented as mean ± SEM.

We examined RvD1 levels using a recently developed ELISA kit (Cayman Chemical). The assay reliably produced the expected RvD1 standard curve (**Figure 2A**). RvD1 levels were measured in serum, spinal cord, and brain tissue samples of naïve mice and mice after sham surgery and bone fracture. Samples were collected on day 5 after surgery, because DHA produced robust analgesic effects at this time point. Interestingly, compared to naïve animals and fracture surgery animals, sham surgery increased RvD1 levels in serum (63.58 pg/ml in naïve and 273.6 pg/ml in sham, *P* < 0.05) but not in brain or spinal cord samples (**Figures 2B–D**), indicating that serum RvD1 level may be correlated with resolution of acute pain in sham animals.

### Perioperative DHA Treatment Increases RvD1 Levels in Serum and Spinal Cord

We then measured RvD1 levels in serum, spinal cord, and brain tissue samples of naïve, sham surgery, and bone fracture mice after vehicle and DHA pre-treatment. Samples were collected at day 5 after surgery as described above. Importantly, RvD1 levels were elevated in serum (271.4 vs. 74.96 pg/ml, *P* < 0.05) and spinal cord samples (1063 vs. 324.4 pg/mg, *P* < 0.05) after systemic DHA pre-treatment (**Figures 2B,D**). There was also a tendency for RvD1 levels to increase in the brain (**Figure 2C**). These data suggest that (1) sham surgery alone, but not bone fracture, can increase RvD1 production or/and its release and (2) DHA might be converted to SPMs such as RvD1 following pre-treatment in this fPOP model.

### Perioperative Treatment of RvD1 and MaR1 Partially Prevents the Development of fPOP

**FIGURE 3:**
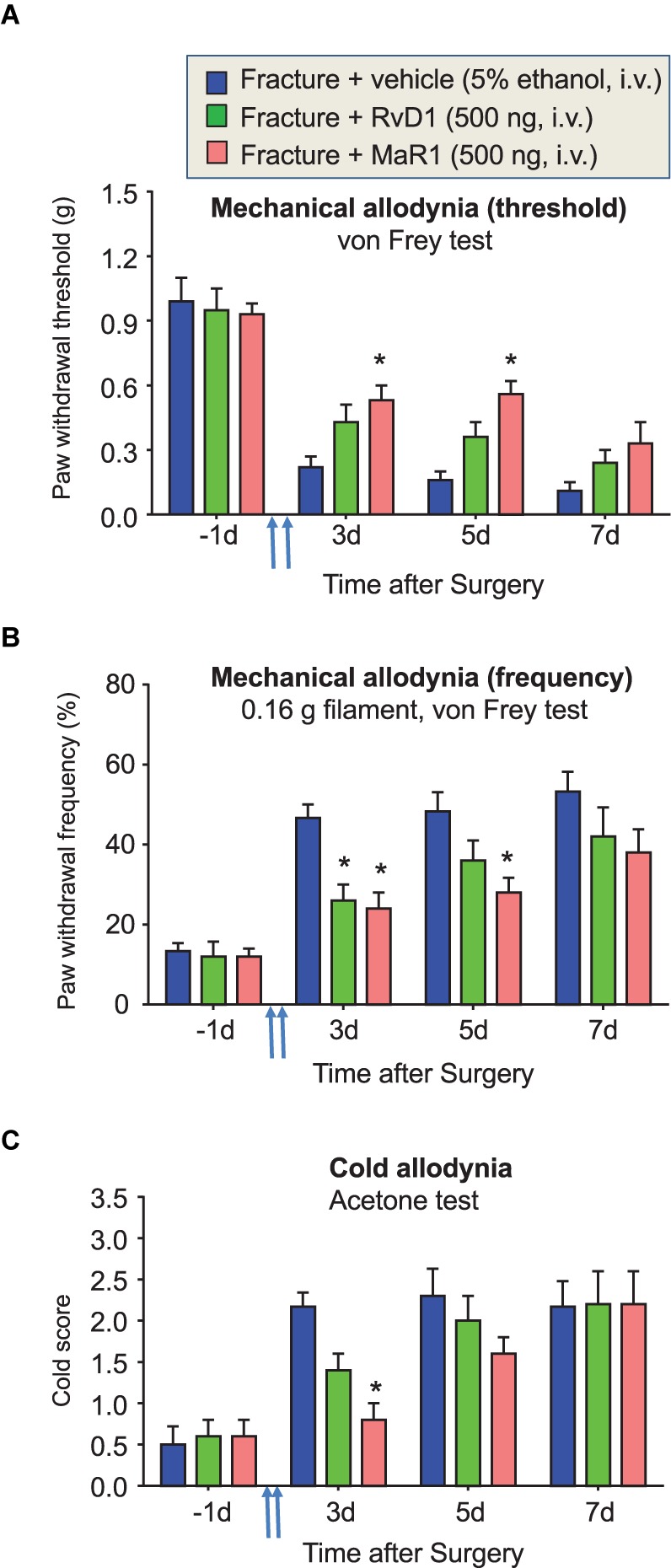
Perioperative treatment of RvD1 and MaR1 attenuates fPOP. Impact of perioperative treatment of RvD1 and MaR1 (500 ng, 100 μl, i.v.), given 10 min and 24 h after bone fracture surgery (indicated by blue arrows), on mechanical allodynia, assessed by paw withdrawal threshold **(A)** and paw withdrawal frequency **(B)** in the von Frey test, as well as on cold allodynia, assessed by cold response scoring in the acetone test **(C)**. ^∗^*P* < 0.05 vs. vehicle (5% ethanol), two-way ANOVA followed by Bonferroni test, *n* = 5 mice/group. Data are presented as mean ± SEM.

RvD1 and MaR1 are DHA-derived SPMs (Serhan et al., 2008). We investigated the potential anti-allodynic effects of RvD1 and MaR1 treatment (500 ng, i.v.) compared to DHA. Notably, RvD1 and MaR1 given at 10 min and 24 h after surgery significantly reduced mechanical and cold allodynia (*P* < 0.05 vs. vehicle). Notably, MaR1 was more effective that RvD1 in reducing allodynia at some time points (**Figures 3A–C**). Thus, SPMs, and especially MaR1, can delay and partially prevent the development of fPOP, at a much lower dose than DHA.

### Spinal Post-operative Treatment of DHA Fails to Inhibit fPOP

**FIGURE 4:**
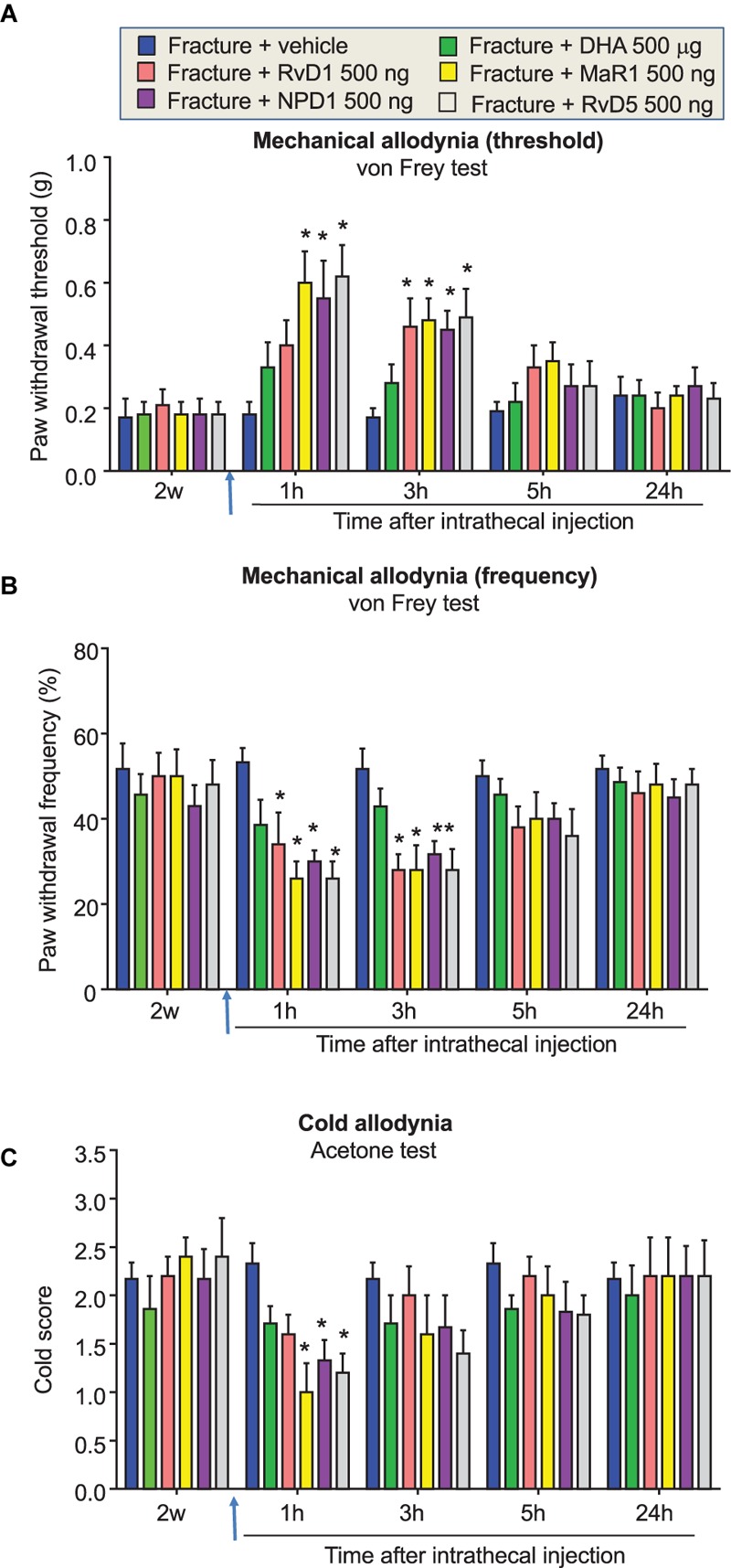
Distinct actions of post-treatment of DHAand DHA-derived SPMs on fPOP. Impact of post-surgical treatment with DHA (500 μg, i.t.) or DHA-derived SPMs RvD1, MaR1, NPD1, and RvD5 (500 ng, 10 μl, i.t.), given 2 weeks after bone fracture surgery (indicated by blue arrows) on mechanical allodynia, assessed by measurements of paw withdrawal threshold **(A)** and paw withdrawal frequency **(B)** in the von Frey test, as well as of cold allodynia, assessed by cold response scoring in the acetone test **(C)**. Note that post-treatment of DHA has no effects on fPOP. ^∗^*P* < 0.05 vs. vehicle (10% ethanol), two-way ANOVA followed by Bonferroni test, *n* = 5–7 mice/group. Data are presented as mean ± SEM.

Spinal post-operative treatment of DHA (10–100 μg) via the IT route was shown to inhibit inflammatory pain following complete Freund’s adjuvant and carrageenan injection, but not neuropathic pain (100 μg) after nerve ligation (Xu et al., 2010, 2013b; Lu et al., 2013). We assessed whether spinal post-treatment of DHA at a higher dose (500 μg, IT) would reduce established fPOP. IT injection of DHA, 2 weeks after surgery, produced no significant inhibition (*P* > 0.05) of fracture-induced mechanical and cold allodynia, compared to vehicle injection, although there was a tendency for inhibition (**Figures 4A–C**). It is noteworthy that vehicle injection (10% ethanol, IT) did not affect mechanical and cold allodynia (**Figures 4A–C**).

### Spinal Post-operative Treatment of DHA-Derived SPMs Differentially Regulates fPOP

Next, we investigated the effects of DHA-derived SPMs, including RvD1, NPD1, and MaR1, since systemic and local applications of these SPMs (10–500 ng) have been shown to inhibit inflammatory and neuropathic pain(Serhan et al., 2008, 2012; Ji et al., 2011; Park et al., 2011a). Spinal post-operative treatment with RvD1, NPD1, and MaR1 (500 ng, IT), given 2 weeks after the surgery, significantly reduced mechanical and cold allodynia (*P* < 0.05 vs. vehicle). Interestingly, NPD1 and MaR1 were more effective than RvD1 in reducing mechanical and cold allodynia at some time points (**Figures 4A–C**).

**FIGURE 5:**
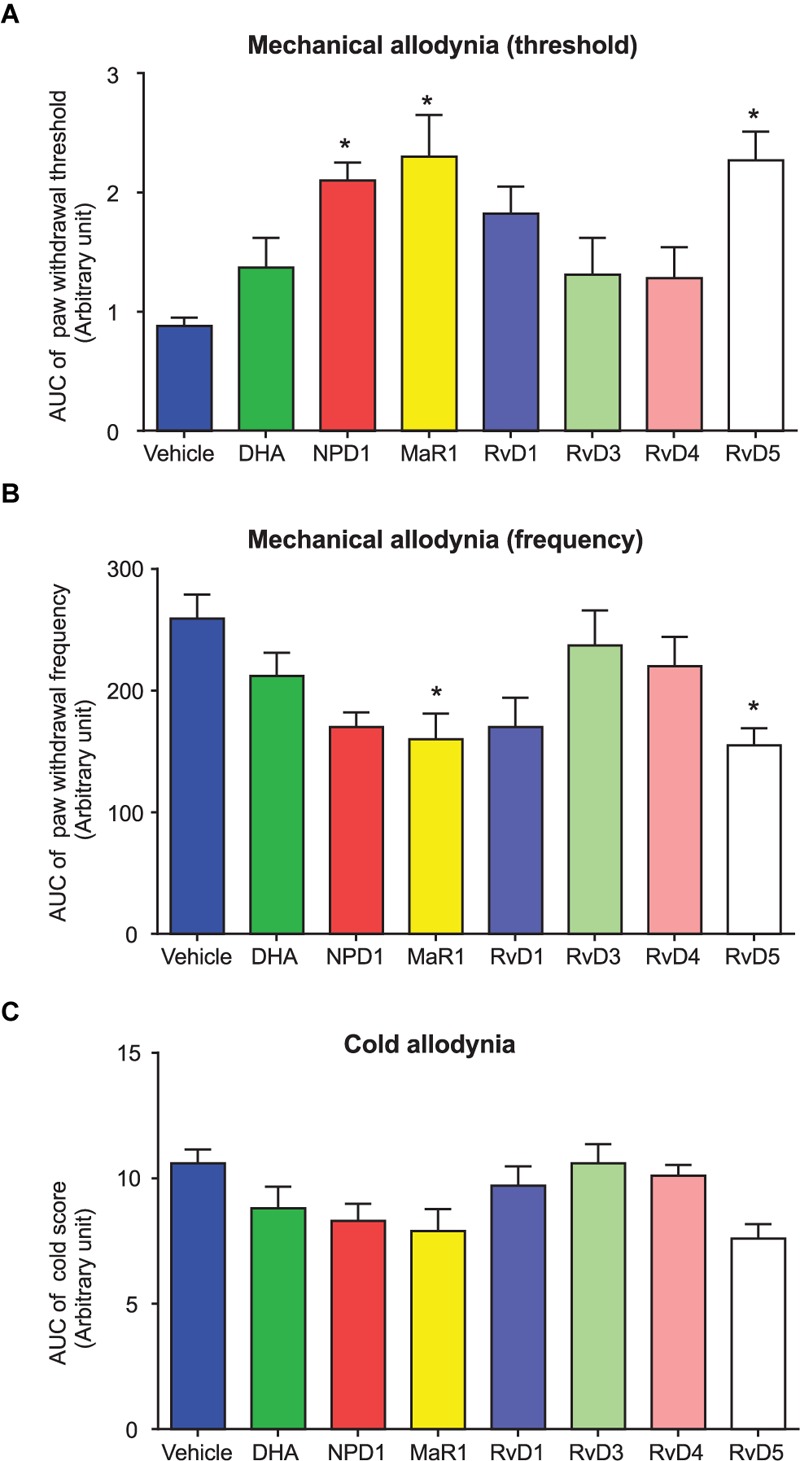
Area under curve (AUC) comparison of distinct post-treatment effects of DHA and DHA-derived SPMs on fPOP. **(A–C)** AUC showing the effects of post-surgical IT treatment of DHA (500 μg, 10 μl) and NPD1, MaR1, RvD1, RvD3, RvD4, and RvD5 (500 ng, 10 μl), given 2 weeks after bone fracture surgery on mechanical and cold allodynia. AUC data were collected at 1–5 h after the drug injection. ^∗^*P* < 0.05 vs. vehicle (10% ethanol), two-way ANOVA followed by Bonferroni test, *n* = 5–7 mice/group. Data are presented as mean ± SEM.

RvD3, RvD4, and RvD5 are newly identified members of resolvin D family and their complete stereochemistry was recently established (Chiang et al., 2012; Arnardottir et al., 2016; Winkler et al., 2016; Norris et al., 2017). However, their effects on pain remained untested. IT injection of RvD5 (500 μg, IT), 2 weeks after surgery, significantly reduced mechanical and cold allodynia (*P* < 0.05, **Figures 4, 5**). By contrast, IT RvD3 and RvD4 (500 ng) did not alter mechanical and cold allodynia (*P* > 0.05, **Figures 5A–C**).

Finally, to further compare the post-operative treatment effects of DHA and DHA-derived SPMs, we plotted all the treatment groups together using area under the curve (AUC) analysis. Because the anti-allodynic effects of SPMs disappeared after 5 h, we only collected AUC data from 1, 3, and 5 h data points after the SPM treatment. One-way ANOVA analysis revealed significant inhibition of mechanical allodynia (**Figures 5A,B**) and cold allodynia (**Figure 5C**) by RvD1, RvD5, NPD1, and MaR1 (*P* < 0.05). RvD5 and MaR1 produced the strongest inhibition of allodynia, whereas DHA only displayed a trend toward inhibition, even at a dose 1000-fold higher than the effective concentrations of the SPMs (**Figures 5A–C**). Taken together, these data demonstrated distinct inhibition of fPOP by post-operative treatment with different SPMs.

## Discussion

There has been a substantial amount of pre-clinical and clinical research on the effects omega-3s (omega-3 unsaturated fatty acids, found in seafood and fish oil) supplements on cardiovascular diseases. However, the findings are inconsistent, as summarized by the National Center for Complementary and Integrative Health, US National Institutes of Health website^1^. For most other conditions for which omega-3s have been studied, definitive conclusions cannot yet be reached. There is more evidence that omega-3s found in seafood and fish oil may help to relieve pain in rheumatoid arthritis (Abdulrazaq et al., 2017). SPMs may provide beneficial effects on cognition and neuroinflammation in aging and Alzheimer’s disease, yet to date clinical studies remain inconclusive (Whittington et al., 2017). Ramsden et al. (2013) demonstrated the effectiveness of omega-3 fatty acid supplementation as a therapy for headache. A follow-up study further showed that targeted alterations in the ratios of dietary omega-3 and omega-6 fatty acids also improved quality of life parameters and reduced psychological distress among patients with chronic headache (Ramsden et al., 2015). Importantly, this study measured plasma concentrations of SPMs [such as RvD2 and the immediate precursors of resolvins and neuroprotectins; 17-hydroxy-docosahexaenoic (17-HDHA) and 18-hydroxy-eicosapentaenoic acid (18-HEPE)] and confirmed that SPMs are converted from dietary omega-3 supplements (Ramsden et al., 2013; Van De Ven and Ji, 2013), suggesting that analgesic effects of fish oil and omega-3-enriched diets may be correlated with the increased production of SPMs. Compared to EPA, DHA is highly enriched in membrane phospholipids of the nervous system and, therefore, plays a more important role in neuroprotection in neurological and neuropsychiatric diseases (Zhang et al., 2014; Liu et al., 2015; Sun et al., 2017). In three large US cohorts, higher circulating levels of DHA are inversely associated with incident atherothrombotic stroke (Saber et al., 2017). Thus, we focused this study on DHA and DHA-derived SPMs. We found that systemic pre-treatment with DHA during the perioperative period (10 min and 24 h after the surgery, 500 μg per mouse, i.v.) alleviated post-surgical pain, especially mechanical hypersensitivity after tibia fracture (**Figure 1**).

Our study also demonstrated that perioperative DHA treatment resulted in increased biosynthesis of RvD1 in serum and in spinal cord (**Figure 2**). Notably, the elevation of RvD1 in the spinal cord was significantly higher than what we measured in the brain on POD5. Especially after the systemic DHA treatment, we found significant increases in RvD1 levels in serum and spinal cord samples but not in brain samples (**Figures 2B–D**). This difference in DHA conversion to RvD1 may result from different activities of the synthesis enzymes such as lipoxygenase (5/15-LOX) in serum and spinal cord vs. brain, since these enzymes are involved in the biosynthesis of RvD1 (Ji et al., 2011). Future studies are needed to examine different regulations of 5/15-LOX expression in blood, spinal cord, and brain tissues after tibial fracture. It is likely that bone fracture causes more upregulations of 5/15-LOX in spinal cord and blood compared to brain. We previously described acute changes (POD1-3) in cognition and glia activation in the brain after orthopedic surgery (Terrando et al., 2011, 2013), suggesting that the resolution of inflammation differs between the brain and the spinal cord. The mechanisms underlying supraspinal effects and communication from the spinal cord to the brain warrant further investigation. In our studies, we also found that perioperative treatment with RvD1 at a much lower dose (500 ng per mouse, i.v.) reduced the development of fPOP, although MaR1 appears to be more effective than RvD1 (**Figure 3**). Further industrial and academic efforts are warranted to optimize ELISA kits for various SPMs without requiring lipidomic platforms. Notably, the effective doses of SPMs in protecting from fPOP in mice are 1000 times lower than for their DHA precursor, as we have previously shown in inflammatory pain conditions (Xu et al., 2010). It is conceivable that the therapeutic effect of perioperative DHA treatment is associated with its conversion to SPMs and these mechanisms warrant further consideration.

Another interesting finding of this study is that sham surgery caused a significant increase in serum RvD1 levels on POD5 compared to naïve control. Importantly, this increase was associated with a transient development of mechanical and cold allodynia that resolved on day 7 (**Figure 1**), suggesting that early SPM production may be responsible for the resolution of acute pain following sham surgery. This is also in agreement with our previous studies as SPMs are synthesized during the resolution phase of acute inflammation (Serhan et al., 2008). Thus, we also propose that a failure in SPMs biosynthesis can drive chronic inflammation (Serhan et al., 2008; Ji et al., 2011). Indeed, RvD1 levels after bone fracture were lower than the levels measured after sham surgery and in naïve animals (**Figure 2**). It is suggested that dysfunction of the biosynthetic pathway of SPM production may result in a transition from acute to chronic POP. While paw incision and muscle retraction produce acute POP (Brennan et al., 1996; Huang et al., 2011), fPOP represents a unique orthopedic surgical model that produces long-lasting POP for many weeks (Wei et al., 2016). In our previous work, we did not find significant changes in mechanical allodynia with opioid analgesia and using a different mouse strain (C57BL/6), suggesting that other factors may influence this response (Zhang et al., 2016). However, as confirmed in this study, the development of cold allodynia is robust and may indicate unique signaling pathways triggered by orthopedic surgery that are consistent with clinical evaluations in post-fracture patients (Serra et al., 2009). It remains to be tested whether SPM or DHA pre-treatment can delay or prevent fPOP. Unlike other POP models, fPOP may manifest as both inflammatory pain and neuropathic pain. In fact, the tibial fracture model used here is regarded as a model for complex regional pain syndrome (Wei et al., 2016). It also produces marked nerve injury, as revealed by increased ATF3 expression in DRG neurons (Zhang et al., 2016), which is reminiscent of ATF3 induction upon thoracotomy that produces long-lasting neuropathic pain (Chi-Fei Wang et al., 2013).

It is noteworthy that post-operative treatment with DHA (500 μg, i.t.), 2 weeks after orthopedic surgery, failed to reduce established fPOP (**Figures 4, 5**). This is in agreement with our previous report that IT DHA (600 μg, given 2 weeks after surgery) did not alter neuropathic pain after nerve ligation (Xu et al., 2013b). However, IT post-treatment of NPD1 was as effective as gabapentin in attenuating nerve injury-induced late-phase mechanical allodynia, despite a striking dose difference (500 ng NPD1 vs. 100 μg gabapentin; Xu et al., 2013b). Despite the effectiveness of the post-operative treatment, we also have to point out that SPMs are more effective in preventing the development of chronic POP and neuropathic pain (Huang et al., 2011; Xu et al., 2013b; Wang and Strichartz, 2017).

Given the rapid expansion of SPMs, it is important to understand distinct roles of SPMs in the resolution of inflammation and pain. We employed AUC analysis to compare the analgesic efficacy of different DHA-derived SPMs following IT post-treatment 2 weeks after orthopedic surgery. It is of interest that among these SPMs MaR1, NPD1, and RvD5 are more effective than RvD1, RvD3, and RvD4 (**Figure 5**). Mechanistically, SPMs resolve acute inflammation by modulating the function of immune cells, such as phagocytosis of macrophages (Serhan et al., 2008). In this study, we evaluated different families of SPMs derived both from the DHA and EPA pathways. These differentially impact G protein-coupled receptor (GPCR) superfamily signaling, which may provide insights into potency and efficacy in different models. Increasing evidence suggests that neuroinflammation, as characterized by activation of glial cells and generation of pro-inflammatory cytokines in the peripheral and central nervous system, plays a critical role in the development and maintenance of chronic pain (Ji et al., 2018). SPMs such as NPD1 and RvE1 control neuroinflammation by inhibiting glial activation and release of TNF and IL-1β in glial cells (Xu et al., 2013a,b). Recent work by Bisicchia et al. (2018) showed that RvD1 also reduces glia activation, both in microglia and astrocytes, and prevented neuronal cell death after remote brain damage. Prophylaxis with aspirin-triggered RvD1 is also effective in protecting the brain from cognitive deficits after surgery by reducing astrocyte activation and neuronal plasticity (Terrando et al., 2013). Importantly, aspirin jumpstarts resolution by generating AT-RvD1 with the 17R configuration of its carbon 17 position, which provides longer lasting *in vivo* effects (Sun et al., 2007). Mimetics of endogenous SPMs, such as targeted nanoparticles, also provide an attractive therapeutic strategy to extend the therapeutic effects and improve delivery of bioactive compounds (Norling et al., 2011; Fredman et al., 2015; Leoni et al., 2015). SPMs also regulate the function of nociceptive neurons in the PNS and of pain circuits within the CNS. For example, RvE1, RvD1, RvD2, and NPD1 inhibit spinal cord synaptic plasticity after inflammation and nerve injury and the function of TRPA1 and TRPV1 ion channels (Xu et al., 2010, 2013b; Park et al., 2011a,b).

## Conclusion

Docosahexaenoic acid and SPMs have distinct potent analgesic actions in preventing and reversing fPOP and chronic pain; for the pretreatment, 1000 times higher amounts of DHA were required than the SPM, which are active in the nanogram range. It is significant and possibly cost saving to implement pro-resolution-derived therapies to prevent the development of chronic neuropathic pain after orthopedic surgery and other conditions, such as chemotherapy, that share similar endpoints. We expect strategies that target endogenous resolution programs to be beneficial in treating several complications within perioperative care and patient’s recovery.

## Author Contributions

LZ, NT, Z-ZX, and SB did the experiments and analyzed the data. S-EJ and WM contributed to project discussion. R-RJ, NT, Z-ZX, and CNS wrote the paper.

## Conflict of Interest Statement

The authors declare that the research was conducted in the absence of any commercial or financial relationships that could be construed as a potential conflict of interest.

## Footnotes

1 https://nccih.nih.gov/health/omega3/introduction.htm

## Abbreviations

MaR1: maresin 1: 7*R*,14*S*-dihydroxy-4*Z*,8*E*,10*E*,12*Z*,16*Z*,19*Z*-docosahexaenoic acid
NPD1: neuroprotectin D1: 10*R*,17*S*-dihydroxy-4*Z*,7*Z*,11*E*,13*E*,15*Z*,19*Z*-docosahexaenoic acid
RvD1: Resolvin D1: 7*S*,8*R*,17 *S*-trihydroxydocosa-4*Z*,9*E*,11*E*, 13*Z*,15*E*,19*Z*-hexaenoic acid
RvD2: Resolvin D2: 7*S*,16*R*,17*S*-trihydroxydocosa-4*Z*,8*E*,10*Z*,12*E*,14*E*,19*Z*-hexaenoic acid
RvD3: Resolvin D3: 4*S*,11*R*,17*S*-trihydroxydocosa-5*Z*,7*E*,9*E*,13*Z*,15*E*,19*Z*-hexaenoic acid
RvD4: Resolvin D4: 4*S*,5*R*,17*S*-trihydroxydocosa-6*E*,8*E*,10*Z*,13*Z*,15*E*,19*Z*-hexaenoic acid
RvD5: Resolvin D5: 7*S*,17*S*-dihydroxy-4*Z*,8*E*,10*Z*,13*Z*,15*E*,19*Z*-docosahexaenoic acid
